# Associations between Self-Reported Gastrointestinal Illness and Water System Characteristics in Community Water Supplies in Rural Alabama: A Cross-Sectional Study

**DOI:** 10.1371/journal.pone.0148102

**Published:** 2016-01-28

**Authors:** Christine E. Stauber, Jessica C. Wedgworth, Pauline Johnson, Julie B. Olson, Tracy Ayers, Mark Elliott, Joe Brown

**Affiliations:** 1 Division of Environmental Health, School of Public Health, Georgia State University, Atlanta, Georgia, United States of America; 2 Department of Biological Sciences, University of Alabama, Tuscaloosa, Alabama, United States of America; 3 Department of Civil, Construction and Environmental Engineering, University of Alabama, Tuscaloosa, Alabama, United States of America; 4 School of Civil and Environmental Engineering, Georgia Institute of Technology, Atlanta, Georgia, United States of America; The Australian National University, AUSTRALIA

## Abstract

**Background:**

Community water supplies in underserved areas of the United States may be associated with increased microbiological contamination and risk of gastrointestinal disease. Microbial and health risks affecting such systems have not been systematically characterized outside outbreak investigations. The objective of the study was to evaluate associations between self-reported gastrointestinal illnesses (GII) and household-level water supply characteristics.

**Methods:**

We conducted a cross-sectional study of water quality, water supply characteristics, and GII in 906 households served by 14 small and medium-sized community water supplies in Alabama’s underserved Black Belt region.

**Results:**

We identified associations between respondent-reported water supply interruption and any symptoms of GII (adjusted odds ratio (aOR): 3.01, 95% confidence interval (CI) = 1.65–5.49), as well as low water pressure and any symptoms of GII (aOR: 4.51, 95% CI = 2.55–7.97). We also identified associations between measured water quality such as lack of total chlorine and any symptoms of GII (aOR: 5.73, 95% CI = 1.09–30.1), and detection of *E*. *coli* in water samples and increased reports of vomiting (aOR: 5.01, 95% CI = 1.62–15.52) or diarrhea (aOR: 7.75, 95% CI = 2.06–29.15).

**Conclusions:**

Increased self-reported GII was associated with key water system characteristics as measured at the point of sampling in a cross-sectional study of small and medium water systems in rural Alabama in 2012 suggesting that these water supplies can contribute to endemic gastro-intestinal disease risks. Future studies should focus on further characterizing and managing microbial risks in systems facing similar challenges.

## Introduction

The burden of gastrointestinal illness (GII) associated with drinking water supplies in the United States (US) is not precisely known [1]. Although available surveillance data suggest declining numbers of outbreaks [2], aging infrastructure and distribution system deficiencies represent persistent challenges that may be associated with increased risks [1,3,4]. Estimates of the endemic attributable disease burden of acute gastroenteritis associated with public water supplies in the US range from 4.3–16.4 million cases annually [5,6], contributing to over 40,000 hospital admissions each year at a cost of at least $970 million [7].

Small water supplies account for the majority of non-compliance with drinking water regulations in the USA [8]. Many also serve rural areas, where operational and financial challenges are prevalent as systems age. Despite the number of these systems and their potential for posing increased risk, there have been no systematic studies of non-outbreak microbial risk in drinking water supplies in underserved, rural areas of the US [4]. As part of a broad assessment of drinking water infrastructure and microbial risks in this setting, we conducted a cross-sectional study of self-reported GII among people served by 14 rural water supplies in Alabama. In 2010, 41% of Alabama’s population was considered rural [9]. The 14 rural water supplies from our study were located in three counties which were >85% rural and comprised a total population of approximately 41,000 people [9]. Our primary goal was to identify reported and measured water system characteristics associated with self-reported GII. Like other rural water supplies, these systems face a range of operational challenges (e.g., low population density and long residence time) and serve a vulnerable, predominantly minority population [4,10,11].

## Materials and Methods

The entire study, including the methods for household recruitment and informed consent, the data analysis plan and publication plan, was reviewed and approved by the Institutional Review Board of the University of Alabama (IRB #10-OR-390-R2). The primary respondent was informed about the study and written consent was provided when the primary respondent agreed to participate. We conducted this study in Alabama’s Black Belt region in 2012, an underserved region characterized in part by high poverty, high unemployment, decreasing population, and high percentage of minorities, especially African-Americans [12]. Other common themes in the region include aging infrastructure and limited access to basic services and health care [13,14]. Problems with water and sanitation infrastructure in the area have been previously documented [11,15–18]. Within the three-county study area, there are 14 water supplies serving 350–10,500 persons, with six classified as small or very small systems (<3,300 persons), and seven medium-sized systems with five serving under 6,600 persons, and two serving >6,600 persons.

We randomly selected households from a master list of consumers provided by water supply utilities and grouped them into geographical areas of ten households to simplify logistics. We visited approximately 2400 households in the region until we reached our target of 300 households per county. When available, the head of the household was informed about the study and asked to participate. If the head of household was not available, another member of the household who was ≥18 years of age was asked to participate. Consenting households were enrolled in the study until the *a priori* sample size criterion of 900 was met. Methods for household recruitment and informed consent were reviewed and approved by the Institutional Review Board of the University of Alabama (IRB #10-OR-390-R2).

Through a survey delivered by trained staff, information on household demographics, socio-economic status, water supply perceptions including delivery and aesthetic characteristics, use and handling of drinking water, and household sanitation was collected. Household level water supply perceptions were assessed as whether or not the primary respondent ever experienced the event (such as low pressure, intermittent service). Individual-level health information was collected for all members of the household by asking the primary respondent about household members’ age, sex and symptoms of GII in the seven days prior to the visit. We used seven-day recall for self-reported symptom data to minimize recall bias [19].

At each household, we collected two water samples from household taps: a flamed sample from the outside tap (if available) and a sample from the kitchen tap as described previously [10]. Water samples were processed within six hours of collection for total coliforms (TC) and *E*. *coli* with IDEXX Colilert^®^ QuantiTrays^®^ (IDEXX Laboratories, Westbrook, Maine). Point-of-sampling water pressure (from outside taps only) was measured with two conforming Rain Bird pressure gauges (Model P2A, Azusa, CA, USA) on a T configuration. Turbidity (Hach 2100Q Portable Turbidimeter, Loveland, CO, USA), free and total chlorine, and pH were measured (Hach Dual Pocket Colorimeter II plus pH, detection limit 0.1mg/L) at the inside sample location.

Data were entered into a Microsoft Access database and transferred to Stata 13 (College Station, Texas) for analysis. To examine associations between reported water service conditions, measured water quality variables and self-reported GII, we performed logistic regression models using the Taylor series linearization method to account for household clusters [20]. We considered multi-level models that addressed water system level data; however, the variance components for water system were not significantly different than zero and thus we reduced the models to accommodate household correlation only. Individual reported health symptoms were classified into one of three categories: any symptoms, any diarrhea, or any vomiting. An individual was classified as having any symptoms if he reported any of the following symptoms in the seven days preceding the survey: watery diarrhea, soft diarrhea, vomiting, nausea, or abdominal cramps. An individual was classified as having diarrhea if the primary respondent reported (for himself or another member of the household) any occurrence of watery and/or soft diarrhea in the seven days preceding the survey while those classified as having vomiting reported any vomiting in the seven days preceding the survey. In addition, we adopted a case definition of acute gastroenteritis (AGI) recommended by Majowicz et al. [21] with some modifications due to limited clinical details. In our study we define a case of AGI as an individual with three or more loose stools or any vomiting in 24 h, but excluding those with irritable bowel syndrome, Crohn’s disease, ulcerative colitis, celiac disease, or another condition with symptoms of diarrhea or vomiting such as pregnancy. All reported water service conditions were examined as dichotomous exposures (such as “experienced low water pressure” versus “did not experience low water pressure”). All water quality measures were also examined as dichotomous exposures (with the exception of pressure) based on *a priori* categories. The following variables were treated as dichotomous: free and total chlorine (absence of chlorine as referent), turbidity (<0.3 NTU as referent), TC and *E*. *coli* (absence as referent). Measured point-of-sampling pressure was log-transformed and examined as a continuous variable.

For the purposes of estimating adjusted Odds Ratios (aORs), we examined socio-demographic and water handling variables that might be associated with the outcomes of interest. The following household level variables were considered for inclusion into the multivariable models: being a rental tenant, presence of college graduates in the home, connection to sewer, reported treatment of tap water, and reported use of bottled water for drinking. We also examined the following individual characteristics: age, race, and report of any chronic or temporary conditions that might be associated with GII such as Crohn’s Disease, Irritable Bowel Syndrome, milk intolerance, and being pregnant. We initially examined associations between each variable and each reported health outcome in univariable logistic regression models. For multivariable model selection, we considered confounders and assessed for changes in effect size estimates of greater than 10%. In addition, any variable that had a significant association (p<0.05) was considered for inclusion in the multivariable models. Variables that were found to be statistically significant were also assessed for interactions.

## Results

Descriptive statistics regarding the participants from the study are presented in Table 1. From February to December 2012, a total of 906 households (composed of 2285 individuals) were recruited. Most owned their homes (92%), 27% were connected to a sewer system while others relied on septic (68%) or did not report any type of treatment for household sanitary waste (5%). Low water pressure was the most frequently reported problem and intermittent service was reported the least frequently. Median age was 46 and 5% of individuals were under 5 years of age. Females were the majority (55%) and 65% were African American. A total of 99 (4.3%) individuals reported experiencing at least one symptom of GII in the seven days preceding the survey, with 37 people (1.6%) reporting vomiting and 43 (1.9%) reporting diarrhea. Thirty-four people (1.5%) reported a chronic or other condition, which would be associated with increased reporting of GI symptoms, which included at least one of the following: Crohn’s Disease, Diverticulitis, Irritable Bowel Syndrome, Ulcerative Colitis, milk intolerance, or pregnancy. Among those with a reported chronic condition, eight reported any symptoms of GII (24%), 3 reported vomiting (9%), and 2 (6%) reported diarrhea. A total of 55 cases of AGI were identified (including three cases of diarrhea where frequency of the stool was unknown). With the exception of any symptoms of GII, these proportions of outcomes were not significantly different than that of those without a chronic condition.

**Table 1:** Characteristics of Households and Participants from a Cross-sectional Study on Water Supply and Health in Rural Alabama 2012.

**Reported household and water service conditions**	**Total Obs N = 906 (%)**
**Median year moved into home**	1997
**Own or buying home**	836 (92.3)
**Sanitation services**	
**Connected to sewer**	6.5)
**Septic system**	598 (68.2)
**Cess pool/pipe to ground**	47 (5.2)
**Household has at least one college graduate**	221 (24.4)
**Has experienced the following with respect to water supply:**	
**Intermittent service**^a^	4.5)
**Low water pressure**^a^	5.4)
**Disagreeable odor**^a^	(17.1)
**Disagreeable taste**^a^	0.9)
**Odd color**^a^	7.1)
**Report drinking bottled water**^b^	445 (49.1)
**Report treating tap water**	149 (16.5)
**Reported individuals characteristics**	**Total observations N = 2285 (%)**
**Age**	Mean Age = 43
**Age distribution**	IQR (21, 62)
**Male**	1032 (45%)
**Individuals reporting race as African-American**	1497 (65%)
**Any symptoms of gastrointestinal illness**^c^	99 (4.3%)
**Diarrhea in the seven days preceding the survey**	37 (1.6%)
**Vomiting in the seven days preceding the survey**	43 (1.9%)
**Reported a chronic or other condition that may impact reports of gastrointestinal illness**^d^	34 (1.5%)

^a^—Household respondent was asked if ever experienced condition

^b^—Household respondent indicate that the household consumed water from bottled water source but frequency not measured

^c^—Individuals reported having at least one the following symptoms in the preceding 7 days: nausea, abdominal cramps, watery diarrhea, soft diarrhea, and/or vomiting

^d^—Conditions included any reports of Crohn’s Disease, Diverticulitis, Irritable Bowel Syndrome, Ulcerative Colitis, milk intolerance, or pregnancy

Water samples did not uniformly meet applicable state or federal standards or guidelines; a summary of the results of water sampling is presented in Table 2. Turbidity and pressure were most frequently outside recommended limits with 42.6% and 25.6% of samples, respectively. The average measured water pressure at the point of sampling was 462 kPa (67 psi), and median turbidity was 0.26 NTU. Almost 14% of households had free chlorine residuals <0.2mg/L, 5% had total chlorine residuals <0.2mg/L and 3.5% and 1.5% of samples had no detectable (<0.1 mg/L) free or total chlorine, respectively. Almost 17% of samples drawn at outside taps were positive for TC compared with 12% of samples taken at kitchen taps, but very few samples were positive for *E*. *coli* (<1%).

**Table 2:** Summary of Measured Water Quality from Households in a Cross-sectional Study on Water Supply and Health in Rural Alabama 2012.

Water Quality Measure (N = 906)	Water quality criterion	% samples meeting applicable standards
**Turbidity (NTU)**	≤0.3^a^	58.4
**Pressure (PSI)**^b^	≥15 & ≤ 80^c^	74.4
**Free chlorine (mg/L)**	≥0.2^d^	86.3
**Total chlorine (mg/L)**	≥0.2^d^	94.4
**Outdoor tap total coliforms (MPN/100mL)**^b^	<1^a^,^d^	83.1
**Kitchen tap total coliforms (MPN/100mL)**	<1^a^,^d^	87.9
**Outdoor tap *E*. *coli* (MPN/100mL)**^b^	<1^a^,^d^	99.7
**Kitchen tap *E*. *coli* (MPN/100mL)**	<1^a^,^d^	99.2

^a^—Guidelines on turbidity recommendations were based on USEPA guidelines for drinking water contaminants[22]

^b^—Pressure was only measured at outdoor taps and outdoor taps were not present at all locations nor did all households with outdoor taps allow for pressure measurement or flame sterilization of tap for bacterial testing. A total of 46 households lacked either a pressure measurement or bacterial measures and 23 households lacked both.

^c^—Guidelines for water pressure were based on the Uniform Plumbing Code[23]

^d^—Guidelines for chlorine residuals and total coliforms and *E*. *coli* were based on the World Health Organization Guidelines for Drinking Water Quality as well as former USEPA requirements of the Surface Water Treatment Rule[24]

A total of five water service conditions and eight water quality parameters were examined for independent associations with the four GII outcomes and the results are presented in Table 3. In the unadjusted analysis, most water service conditions were found to be associated with all four GII conditions although odd color was not associated with vomiting. Five water quality measures were statistically significantly associated with at least one GII outcome measure: log_10_ pressure, turbidity, absence of free chlorine or total chlorine and the presence of *E*. *coli* in (flamed) samples from outside tap.

**Table 3:** Unadjusted Associations between Reported Water Service Conditions, Measured Water Quality and Reported Health Outcomes in a Cross-sectional Survey of Households in Rural Alabama 2012.

Exposure	Any symptoms	Any vomiting	Any diarrhea	AGI^b^
	OR (95%CI)^a^	OR (95%CI)^a^	OR (95%CI)^a^	OR (95%CI)^a^
**Intermittent service**	3.12 (1.79–5.44)*	3.39 (1.44–7.99)*	7.76 (3.24–18.56)*	3.78 (1.83–7.78)*
**Low water pressure**	4.79 (2.81–8.17)*	7.81 (2.99–20.42)*	7.70 (2.97–19.94)*	5.54 (2.64–11.65)*
**Displeasing taste**	2.65 (1.52–4.61)*	3.52 (1.51–8.26)*	4.90 (2.01–11.93)*	3.14 (1.50–6.56)*
**Displeasing odor**	3.09 (1.77–5.41)*	2.92 (1.20–7.13)*	6.25 (2.59–15.17)*	4.07 (1.98–8.37)*
**Odd color**	2.09 (1.19–3.67)*	1.65 (0.70–3.88)	4.00 (1.67–9.58)*	2.48 (1.23–5.01)*
**Log**_**10**_ **pressure**	0.24 (0.04–1.30)	0.08 (0.007–0.73)*	0.13 (0.005–3.00)	0.13 (0.01–1.13)
**Turbidity >0.3 NTU**	0.75 (0.46–1.26)	0.38 (0.17–0.86)*	0.70 (0.29–1.67)	0.77 (0.39–1.52)
**Free chlorine absent**	3.72 (1.23–11.29)*	6.70 (1.77–25.31)*	4.41 (0.80–24.11)	4.19 (0.97–18.11)
**Total chlorine absent**	5.15 (1.21–21.86)*	10.40 (1.84–58.89)*	9.27 (1.13–76.11)*	6.30 (0.78–50.89)
**Outside tap positive for total coliforms**	1.20 (0.62–2.34)	0.74 (0.26–2.06)	0.95 (0.30–2.98)	1.06 (0.44–2.53)
**Kitchen tap positive for total coliforms**	1.44 (0.66–3.14)	1.83 (0.57–5.93)	2.23 (0.70–7.09)	2.13 (0.84–5.37)
**Outside tap positive for *E*. *coli***	2.71 (0.58–12.61)	6.36 (1.32–30.54)*	7.89 (1.62–38.47)*	5.04 (1.06–23.86)*
**Kitchen tap positive for *E*. *coli***	1.16 (0.19–7.23)	2.79 (0.44–17.80)	3.26 (0.51–21.00)	2.25 (0.36–14.00)

^a^–Odds ratios and 95% confidence intervals calculated using Taylor Series Linearization

^b^–AGI is defined as an individual with three or more loose stools, or any vomiting, in 24 h, but excluding those with irritable bowel syndrome, Crohn’s disease, ulcerative colitis, celiac disease, or another condition with symptoms of diarrhea or vomiting such as pregnancy

* statistically significant associations (p <0.05)

To address potential confounding, we examined associations between self-reported GII and socio-demographic variables. These results are presented in Table 4. Two variables (bottled water use and reporting a chronic disease) were associated with all four of the GII outcomes and one (report of treating tap water) was associated with any symptoms of GII (but not the other three). Since these two variables remained significant and impacted the effect size of our water quality estimates, they were retained in multivariable models. After an analysis of interaction, reporting a chronic condition was also found to be an effect modifier. However, due to limited sample size (only 34 individuals with this condition), we were unable to provide stratified estimates for this group as a result of quasi-complete data separation and Maximum Likelihood Estimates could not be computed. As a result, the final models include reported treatment of tap water and use of bottled water and exclude observations from participants who reported having a chronic condition. The unadjusted results are presented in Table 3 and the adjusted results are presented in Table 5.

**Table 4:** Unadjusted Associations between Socio-demographic, Individual and Water Handling Characteristics and Reported Health Outcomes in a Cross-sectional Survey of Households in Rural Alabama in 2012.

Variable	Any symptoms	Any Vomiting	Any Diarrhea	AGI^b^
	OR (95%CI)^a^	OR (95%CI)^a^	OR (95%CI)^a^	OR (95%CI)^a^
**Rents home**	0.68 (0.25–1.87)	0.38 (0.05–2.78)	0.95 (0.22–4.14)	0.62 (0.15–2.60)
**One or more college grads in the home**	1.02 (0.58–1.83)	0.94 (0.37–2.39)	0.83 (0.28–2.42)	0.84 (0.36–1.92)
**Connected to sewer**	0.79 (0.43–1.45)	0.67 (0.24–1.87)	1.07 (0.41–2.77)	0.92 (0.42–2.02)
**Report drinking bottled water**	2.19 (1.31–3.62)*	2.71 (1.20–6.12)*	2.51 (1.09–5.78)*	2.10 (1.07–4.14)*
**Report treating tap water**	1.98 (1.08–3.64)*	1.79 (0.68–4.68)	1.14 (0.44–2.94)	1.63 (0.71–2.37)
**Age (continuous)**	0.99 (0.99–1.01)	0.98 (0.97–0.99)	1.00 (0.99–1.01)	
**Age binary (<5 as referent)**	1.33 (0.39–4.51)	0.53 (0.16–1.78)	0.97 (0.14–6.53)	0.70 (0.21–2.37)
**Race (binary)**	0.85 (0.49–1.45)	2.26 (0.86–5.96)	0.81 (0.34–1.95)	1.27 (0.59–2.69)
**Sex (male as referent)**	1.44 (0.96–2.16)	1.18 (0.63–2.21)	0.89 (0.50–1.59)	1.11 (0.65–1.89)
**Respondent reported illness that may increase GII**^c^	7.30 (3.06–17.40)*	5.35 (1.49–19.15)*	3.96 (0.87–17.96)	N/A

^a^—Odds ratios and 95% confidence intervals calculated using Taylor Series Linearization

^b^—AGI is defined as an individual with three or more loose stools, or any vomiting, in 24 h, but excluding those with irritable bowel syndrome, Crohn’s disease, ulcerative colitis, celiac disease, or another condition with symptoms of diarrhea or vomiting such as pregnancy

^c^—Conditions included any reports of Crohn’s Disease, Diverticulitis, Irritable Bowel Syndrome, Ulcerative Colitis, milk intolerance, or pregnancy

* statistically significant associations (p <0.05)

**Table 5:** Multivariable Associations between Reported Water Service Conditions, Measured Water Quality and Reported Health Outcomes in a Cross-Sectional Survey of Households in Rural Alabama in 2012.

Exposure	Any symptoms	Any vomiting	Any diarrhea	AGI^b^
	adjusted OR (95%CI)^a^	adjusted OR (95%CI)^a^	adjusted OR (95%CI)^a^	adjusted OR (95%CI)^a^
**Report of intermittent service**	3.01 (1.65–5.49)*	2.47 (0.96–6.38)	6.23 (2.44–15.93)*	3.11 (1.49–6.50)*
**Report of low water pressure**	4.51 (2.55–7.97)*	7.18 (2.73–18.91)*	6.31 (2.56–15.61)*	5.04 (2.54–9.97)*
**Report of displeasing taste**	1.83 (0.92–3.66)	1.80 (0.62–5.25)	3.23 (1.17–8.93)*	2.31 (1.00–5.35)*
**Report of displeasing odor**	2.55 (1.33–4.91)*	1.95 (0.68–5.58)	4.97 (1.85–13.37)*	3.05 (1.37–6.79)*
**Report of odd color**	1.71 (0.89–3.28)	0.90 (0.35–2.32)	2.84 (1.04–7.79)*	1.89 (0.89–4.03)
**Log**_**10**_ **Pressure**	0.21 (0.03–1.61)	0.05 (0.003–0.57)*	0.09 (0.002–3.21)	0.11 (0.01–1.14)
**Turbidity >0.3 NTU**	0.74 (0.42–1.31)	0.47 (0.20–1.10)	0.92 (0.36–2.32)	0.85 (0.42–1.71)
**Free chlorine absent**	3.57 (1.13–11.22)*	6.39 (1.46–27.89)*	5.33 (0.94–30.37)	4.17 (0.99–17.44)
**Total chlorine absent**	5.73 (1.09–30.13)*	11.07 (1.37–89.23)*	11.96 (1.50–95.38)*	7.29 (0.93–57.07)
**Outside tap positive for total coliforms**	1.21(0.58–2.51)	0.72(0.24–2.23)	0.93 (0.26–3.31)	1.03 (0.42–2.54)
**Kitchen tap positive for total coliforms**	1.80 (0.80–4.05)	2.40 (0.70–8.21)	2.98 (0.89–9.94)	2.43 (0.93–6.35)
**Outside tap positive for *E*. *coli***	2.02 (0.76–5.40)	5.01 (1.62–15.54)*	7.75 (2.06–29.15)*	3.79 (1.25–11.47)*
**Kitchen tap positive for *E*. *coli***	0.92 (0.18–4.65)	2.26 (0.42–12.21)	3.40 (0.57–20.34)	1.71 (0.32–9.16)

^a^–Odds ratios and 95% confidence intervals are adjusted for reported treatment of tap water and drinking bottled water (excludes 34 observations with any chronic illnesses reported) and are calculated using Taylor Series Linearization

^b^–AGI is defined as an individual with three or more loose stools or any vomiting in 24 h, but excluding those with irritable bowel syndrome, Crohn’s disease, ulcerative colitis, celiac disease, or another condition with symptoms of diarrhea or vomiting such as pregnancy

* statistically significant associations (p <0.05)

After adjusting for reported water handling practices of treating tap water and drinking bottled water, participants that reported experiencing low water pressure had a 4.5 times (aOR: 4.51 (95% CI 2.55–7.97)) higher odds of reporting any symptoms of GII and five to seven times higher odds of reporting AGI (aOR: 5.04 (2.54–9.97)), diarrhea (aOR: 6.32 (95% CI 2.56–15.61)) or vomiting (aOR: 7.18 (95% CI 2.73–18.91)), respectively. Those who reported intermittent service had three times a higher odds of reporting any symptoms (aOR: 3.0 (95% CI 1.65–5.49)) or AGI (aOR: 3.11 (1.49–6.50)) and six times higher odds of reporting diarrhea (aOR: 6.2 (95%CI 2.44–15.93)). Vomiting was not statistically significant in the adjusted model.

After adjusting for reported treatment of tap water and drinking bottled water, reports of displeasing taste, odor and color all remained statistically significantly associated with increased odds of diarrheal disease and reported taste and odor problems were also associated with AGI in the adjusted model but none were associated with vomiting. Participants that reported displeasing taste, odor or odd color had 2to 5 times increased odds of diarrhea or AGI compared to households that did not experience these aesthetic problems. Participants that reported displeasing odor had 2.5 times increased odds for any symptoms of GII (aOR: 2.55 (95% CI 1.33–4.91)).

Four measured water quality variables maintained statistically significant associations with self-reported GII in the adjusted models. The absence of total chlorine was associated with an increased odds of reporting any symptoms of GII (aOR: 5.73 (95% CI 1.09–30.13)), vomiting (aOR: 11.07 (95% CI 1.37–89.23)) or diarrhea (aOR: 11.96 (95% CI 1.50–95.38)). The absence of free chlorine residual was also found to be associated with increased odds of reporting vomiting and any symptoms of GII and was marginally associated with AGI but not diarrheal disease. Detection of *E*. *coli* was associated with increased odds of reporting vomiting (aOR: 5.01 (95% CI 1.62–15.52)), diarrhea (aOR: 7.75 (95% CI 2.06–29.15)), and AGI (aOR: 3.79 (1.25–11.47)) but was not associated with the more general category of any symptoms of GII. The continuous variable of log transformed pressure remained statistically associated with decreased odds of vomiting in the adjusted model. However, aORs’ 95% confidence intervals remained very wide for all of these estimates.

## Discussion

A recent systematic review concluded that water distribution system deficiencies, including temporary water outages, are associated with statistically significant increases in GII [3]. Within water supplies, poor operation and maintenance, aging infrastructure, inadequate treatment, and interrupted or intermittent supply may be associated with increased risks to consumers, especially those who are more vulnerable to waterborne diseases, such as people living with HIV or the elderly [1,2,4,25]. Our study yielded broadly consistent findings. We found that individuals within households reporting problems with water supply such as intermittent service or low water pressure were more likely to report GII in the week preceding the survey; associations which remained statistically significant after adjusting for water handling practices in the home. While we do not have water utility confirmation of water main breaks or transient pressure in the systems, we have some evidence that households that reported decreased water pressure were experiencing it based on our pressure measurements [10]. In our analysis of associations between perceived and measured quality, we found that consumer-reported data for this parameter was generally reliable [10]. Low water pressure and intermittent service provide opportunities for contaminant intrusion [26], resulting in microbial contamination and potentially increased risk for GII. Recent evidence from a documented water emergency in Alabama also found an association between self-reported GII and loss of water pressure and water service [27].

We documented an association between households that reported displeasing aesthetic characteristics and diarrheal disease. While consumer preference and perception of water quality has been frequently measured, especially with respect to purchasing of bottled water, there is a paucity of data surrounding whether or not these perceptions are associated with increased reports of illness, especially in the US. In a study of small water supplies in Oregon, Anadu et al [28] found that communities perceived more risk from their water systems when they were known to violate drinking water standards. In a study of water quality and risk in Europe, researchers found that consumer estimation of quality and risk were strongly influenced by organoleptic (aesthetic) aspects of drinking water [29].

We found that lack of chlorine and presence of *E*. *coli* in samples drawn from outside (flame-sterilized) taps, although infrequent, were associated with reports of GII, consistent with a smaller pilot study in this same area identifying an association between fecal coliform and reported GII [11]. In a cross-sectional survey in Russia, Egorov et al. [30] found that decreases in chlorine residuals in the distribution system were associated with increased self-report of illnesses. We also found that increased pressure (measured at the tap) was associated with decreased reports of vomiting. Researchers documented associations between reported low water pressure and GII in an analysis of data from a case-control study in Europe [31]. We found no association between GII and turbidity, consistent with one previous study that did not identify an association between treated water turbidity and GII captured in emergency department visits in urban Atlanta [32].

This study had several important limitations. As a cross-sectional study, we examine associations between potential exposures and outcomes at a single time point. Although we directly measure standard fecal indicator bacteria in household water, these samples may not be reflective of water quality in the days preceding the survey, when infections detected at the time of the survey would have occurred. Also, our data were limited to indicators of system functionality and potential exposures as measured at the household level; identification of environmental sources of microbial contamination was outside the scope of the study. Additionally, no water supply serving enrolled households had a validated hydraulic model at the time of the study; details on system function, treatment interruption, pressure fluctuations, water age, potential cross-connections, maintenance schedules, and other potentially important infrastructure data were not available for these small, rural supplies, where record-keeping can be basic. Finally, we rely on self-report for health outcomes as well as key exposure variables. Although the informed consent and other scripts were carefully written and delivered to avoid introducing observer bias, self-reported data that cannot be verified independently should always be interpreted with caution. This is particularly true for diarrheal disease, which may be unreliable due to unblinded interventions or exposures leading to reporting bias or recall bias for retrospective symptomology [19]. Further, respondents may not always be well informed about GII symptoms for other members of the household, especially other adults. Access to health care is limited in this underserved region [33] and other, potentially useful triangulating health data were unavailable at the time of study, a limitation noted in other studies of waterborne disease risk from similar settings [34,35] including from rural Alabama [27]. In another study from the same dataset [10], we reported that water pressure data were consistent between self-report and as-measured using pressure gauges at the time of sampling, though independent verification for other important measures we report are unavailable.

Despite limitations of the current analysis, our findings suggest that rural, small and medium-sized community water supplies in underserved settings can contribute to endemic GII risk. Other studies of waterborne disease risk where comparable challenges apply would be helpful, given current unknowns around the distribution and magnitude of the burden, especially in small water supplies [1,5,6,34–37]

## Supporting Information

S1 File Cross-sectional Study Questionnaire. (DOCX)

S2 File Cross-sectional Study Data Set. (DTA)

S1 Table Description of variables used in analysis. (DOCX)
