# Supplementary material for: Associations between Self-Reported Gastrointestinal Illness and Water System Characteristics in Community Water Supplies in Rural Alabama: A Cross-Sectional Study
Source: PLoS One. 2016 Jan 28;11(1):e0148102. doi: 10.1371/journal.pone.0148102 (PMC4731071; doi:10.1371/journal.pone.0148102)
Supplement: S1 File — (DOCX) [file pone.0148102.s001.docx]

**Water Infrastructure Sustainability and Health in Alabama’s Black Belt**

**Enrollment Interview**

| GPS Coordinates : |  | Household Number : |  |
| --- | --- | --- | --- |
| Water System Code : |  | County Number : |  |
| Interviewer Code : |  | Visit Number : |  |

**Do you receive and pay a water bill?**

**[00]-No (If no, terminate interview)**

**[01]-Yes (If yes, proceed to consent)**

**A. CONSENT:**

***[Please read to respondent before beginning the survey. If the respondent does not agree to participate, do not continue the interview]***

Hello! My name is ______________. I am working for a research institution called the University of Alabama, and you are being asked to take part in a research study. The study is called “Water Infrastructure Sustainability and Health in Alabama’s Black Belt.” The study is being paid for by a grant from the Environmental Protection Agency. The University of Alabama and it’s community partner are working together to improve the water quality and health in 3 Alabama counties. We want to know if the water you are receiving into your home is causing disease in your community and in all of the communities involved. Some residents in rural Alabama, have reported problems with water delivery systems. Some people have complained of outages, connection problems, high cost, and unclean water. It is a concern that potentially unsafe drinking water is causing disease. The purpose of our study is to better understand whether water quality is a problem for your health and the health of your community.

You are one of 900 households randomly chosen from 3 Alabama Counties. You have been asked to take part in this study because you are a resident of one of the included counties and your household is connected to a small water supply system. If you agree to participate in this study, we will visit you 4 times, now and once every 6 months, over the next 18 months. You will be asked to complete a survey on water system data and on your family’s water use and health. You will be asked to fill out weekly health diaries to report symptoms of disease and you will be reminded weekly by the research team. You will be asked to allow the research team to do an evaluation of your sanitation system and take photographs of the system. You will also be asked to submit a water sample during the visit. We will ask for one water sample at each visit. We will provide the containers to collect the water samples and we will collect the samples for you. The first visit, today, will take approximately 1 hour, and the remaining visits will take 30 minutes. I will not tell anyone about your answers to these questions. Although we will ask for some personal information about you, such as your name and address, we will never use that information in any report we write. We would like to have that information so we can visit you again in the future.

We do not anticipate any risks or discomfort to you by participating. Please ask me to explain anything that you don’t understand before you make your decision as to whether to participate in the survey or not. Your participation in the study is completely voluntary. You do not have to answer all questions and you can stop your participation at any time. Just let me know at any time if you do not want to answer a question. There is no direct, immediate, and tangible benefit of participating in this survey although we will provide you with the results of the testing of your water sample which might be helpful in learning more about your water quality. If you have questions about this study right now, please ask them. If you have questions about this study later on, please call the project manager (JJ Wedgworth) at (205)-499-0458, or the Principal Investigator, Dr. Pauline Johnson, Department of Civil, Construction, Environmental Engineering, at (205)-348-0418. If you have any questions about your rights as a research participant you many contact Ms. Tanta Myles, The University of Alabama Research Compliance Officer, at (205)-348-5152.

1. Are you willing to complete this interview and participate in this study?

[0] No **(Interviewer: Thank you! Terminate the interview.)**

[1] Yes **(Interviewer: Ask them to sign the informed consent)**

2. Are you willing to allow us to take photographs of your sanitation system and use them for research purposes only?

[0] No

[1] Yes **(Interviewer: Ask them to sign appropriately on informed consent)**

3. Will you allow us to contact you with weekly reminders to fill out your health diaries?

[0] No, (**If No, Skip to 5)**

[1] Yes, **(Interviewer: Ask them to sign appropriately on informed consent)**

4. What way would you prefer us to contact you?

[0] Text Message (Number :____________________________)

[1] Email (Email Address :_______________________)

[2] Phone Call (Number :____________________________)

| **Household Questionnaire Baseline**  ***(To be administered by study personnel to an adult 18 years or older member of the household)*** | | |
| --- | --- | --- |
| 5. Day/Month/Year of Interview: | Day Month Year |  |
| 6. Time of Interview: | Hour Min AM/PM |  |
| 7. Street Address: | Address:____________________________  ___________________________________ |  |
| 8. Mailing Address (If Different)  (**Be Sure to Ask, “Where do you get your mail.”)** | Address:____________________________  ___________________________________  Same as street address |  |
| 9. County | 0=County 1  1=County 2  2=County 3 |  |
| 10. Home Telephone Number: | 99=No Response |  |
| 11. Alternative Telephone Number: | 99=No Reponse |  |
| 12. What is the name of the head of the household? | Name: ____________________________  99=No Response |  |
| 13. **(Observation Only)** Is the head of the household male or female? | 0=Male  1=Female |  |
| 14. Head of household age: | Years  99=No Response |  |
| 15. How many members live in this household? | 99=No Response |  |

| **Water System/Infrastructure Survey Baseline**  ***“Now I would like to ask you a few questions about your water service.”*** | | |
| --- | --- | --- |
| 16. Generally, how would you rate the water pressure in your household?  **(Read all the options and circle one)** | 0=Very Strong  1=Strong  2=Moderate  3=Weak  4=Very Weak  99=No Response |  |
| 17. Have you noticed any of the following issues regarding your water pipes? **(Read all responses and mark 01, 00 or 99 for each choice)** | 00=No, 01=Yes, 99 =Don’t know/no response  a. Leaking Pipes  b. Corrosion on Pipes  c. Frequent Knocking of Pipes  d. Other,  Explain:_________________________ |  |
| 18. Do you ever experience intermittent service or service outages? **(Circle one answer)** | O = No  1 = Yes  99 = Don't know/no response | If 0 or 99, skip to 20 |
| 19. If yes, how often do you have outages or intermittent service? **(Read all the options and circle only one)** | 0=Less than once in six months  1=At least once per month  2=At least once per week  3=Daily  99=Don't know/ no response |  |
| 20. Do you ever experience low water pressure? **(Circle one)** | O = No  1 = Yes  99 = Don't know/no response | If 0 or 99, skip to 22 |
| 21. If yes, how often do you experience low water pressure? **(Read all the options and circle only one)** | 0=Less than once in six months  1=At least once per month  2=At least once per week  3=Daily  99=Don't know/ no response |  |
| 22. Do you ever feel that the water has a bad smell? **(Circle one)** | O = No  1 = Yes  99 = Don't know/no response | If 0 or 99, skip to 24 |
| 23. If yes, how often do you notice this foul/bad smell? **(Read all the options and circle only one)** | 0 Less than once in six months  1 At least once per month  2 At least once per week  3 Daily  99 Don't know/ no response |  |
| 24. Do you ever feel that the water has a bad taste? **(Circle one)** | O = No  1 = Yes  99 = Don't know/no response | If 0 or 99, skip to 26 |
| 25. If yes, how often do you notice this bad taste? **(Read all the options and circle only one)** | 0=Less than once in six months  1=At least once per month  2=At least once per week  3=Daily  99=Don't know/ no response |  |
| 26. Do you ever feel that the water has an odd color to it? **(Circle one)** | O = No  1 = Yes  99 = Don't know/no response | If 0 or 99, skip to 28 |
| 27. If yes, how often do you notice this odd color to the water? **(Read all the options and circle only one)** | 0=Less than once in six months  1=At least once per month  2=At least once per week  3=Daily  99=Don't know/ no response |  |
| 28. Have you experienced any other problems or do you have any other concerns about the water from your pipes? | Please describe: ______________________  ___________________________________ |  |

| **Individual Household Members**  **(Record the following information for all members of the household, include all adults and children)** | | | | | | |
| --- | --- | --- | --- | --- | --- | --- |
| **#** | **29.**  **Head of**  **Household**  **(0=No,**  **1=Yes)** | **30.**  **Name** | **31.**  **Age** | **32.**  **Sex**  **(0=M**  **1=F)** | **33.**  **Race**  **(0=White, 1=Black or African American, 2=American Indian or Alaskan Native, 3=Asian, 4=Native Hawaiian or Pacific Islander,5=Hispanic or Latino, 6=Other)** | **34.**  **Relationship To Head of Household**  **(0=Head of Household, 1=Spouse, 2=Child, 3=Parent, 4=Grandparent, 5 Sibling, 6 = Aunt, 7=Uncle , 8=Cousin, 9=Other Relation, 10=No Relation)** |
| 01-Respondent |  |  |  |  |  |  |
| 02 |  |  |  |  |  |  |
| 03 |  |  |  |  |  |  |
| 04 |  |  |  |  |  |  |
| 05 |  |  |  |  |  |  |
| 06 |  |  |  |  |  |  |
| 07 |  |  |  |  |  |  |
| 08 |  |  |  |  |  |  |
| 09 |  |  |  |  |  |  |
| 10 |  |  |  |  |  |  |
| 11 |  |  |  |  |  |  |
| 12 |  |  |  |  |  |  |

**(For any child listed above ≤ 2 years of age, ask question 35, if not, SKIP TO 36)**

| 35. Which water do you use for baby’s formula?  **(Read all responses and mark 01, 00 or 99 for each choice)** | 00=No,0=Yes, 99= Don't know/No Response  a. breast milk  b. formula using cold tap water  c. formula using heated tap water  d. formula using bottled water |  |
| --- | --- | --- |

| **Individual Health Data**  **“In the past 7 days have you experienced any of the following? “(00=No, 01=Yes,99=Don’t Know/No Response)** | | | | | | | | | |
| --- | --- | --- | --- | --- | --- | --- | --- | --- | --- |
| # | 36.  Watery  Diarrhea | 37.  Soft  Diarrhea | 38. **If yes to diarrhea, how many times did they go to the bathroom in one day?** | 39.  Abdominal  Cramps | 40.  Nausea | 41. Vomiting | 42.  Fever | 43. Pregnant | 44.  Nursing |
| 01 |  |  |  |  |  |  |  |  |  |
| 02 |  |  |  |  |  |  |  |  |  |
| 03 |  |  |  |  |  |  |  |  |  |
| 04 |  |  |  |  |  |  |  |  |  |
| 05 |  |  |  |  |  |  |  |  |  |
| 06 |  |  |  |  |  |  |  |  |  |
| 07 |  |  |  |  |  |  |  |  |  |
| 08 |  |  |  |  |  |  |  |  |  |
| 09 |  |  |  |  |  |  |  |  |  |
| 10 |  |  |  |  |  |  |  |  |  |
| 11 |  |  |  |  |  |  |  |  |  |

| **Do you have any of the following medical conditions? (00=No, 01=Yes, 99=Don’t Know/No Response)** | | | | | | | | |
| --- | --- | --- | --- | --- | --- | --- | --- | --- |
| # | 45.  Crohn’s  Disease | 46.  Diverticulitis | 47.  Irritable  Bowel Syndrome | 48.  Milk  Intolerance | 49.  Stomach  Ulcer | 50. Ulcerative  Colitis | 51.  Migraine  Headache | 52.  Frequent  Heartburn |
| 01 |  |  |  |  |  |  |  |  |
| 02 |  |  |  |  |  |  |  |  |
| 03 |  |  |  |  |  |  |  |  |
| 04 |  |  |  |  |  |  |  |  |
| 05 |  |  |  |  |  |  |  |  |
| 06 |  |  |  |  |  |  |  |  |
| 07 |  |  |  |  |  |  |  |  |
| 08 |  |  |  |  |  |  |  |  |
| 09 |  |  |  |  |  |  |  |  |
| 10 |  |  |  |  |  |  |  |  |
| 11 |  |  |  |  |  |  |  |  |

| 53. When did you move into your home? | Month:____________  Year:______________  99=Don’t Know/No Response |  |
| --- | --- | --- |
| 54. Do you own or are you currently buying your home, or do you rent?**(Circle one)** | 0=Own/Currently Buying  1=Rent  99=Don’t Know/No Response |  |
| 55. Are there any college graduates in the household?**(Read all the options and circle only 1 answer)** | 0=None  1=1  2=2  3=3 or more  99=Don’t Know/No Response |  |
| 56. What is the approximate household income per year, including everyone who normally lives here?**(Do not read, circle one)** | 0=$0-$12,000  1=$12,000-$15,000  2=$15,000- $17,000  3=$17,000-$22,000  4=$22,000-$26,000  5=$26,000-$30,000  6=$30,000- $45,000  7-$45,000-$60,000  8=$60,000-$75,000  9=Above $75,000  99=Don’t Know/No Response |  |
| 57. Do you get your drinking water from a community water system (city or county), private well, or a combination?**(Read all the options and circle only one)** | 0=Community System  1=Private Well  2=Community System and Private Well  99=Don’t Know/No Response |  |
| 58. Do you drink water from any other source except your tap?**(Circle one)** | 0=No  1=Yes  99=Don’t Know/No Response | If 0 Skip to 60 |
| 59. Do you regularly drink water from any of the following other sources? If Yes, Rank the importance of each source? **(Place a 1 in front of the source the participant said he used most, 2 in second most important source, etc. Place a zero if not mentioned).** | 00=No,01=Yes, 99=Don’t Know/No Response  a. Bottled Water  b. Water from workplace  c. Water from a relatives/friend's home  d. Water from a nearby store’s tap |  |

| 60. Does your household treat your tap water used for drinking in any way? | 0 = No  1 = Yes  99=Don’t Know/No Response | If 0,99 Skip to 64 |
| --- | --- | --- |
| 61. What is the most common method that your household uses to treat your water?  **(Read all the options and circle only one)** | 0=Brita Filter  1=Tap Filter  2=Disinfection by Boiling  3=Other, Method:__________________  99=No Response |  |
| 62. What is the main reason that you treat your tap water?**(Read all the options and circle only one)** | 0=Smell  1=Taste  2=Color  3=Clarity  4=Doctor Recommendation  5=Health Problems  6=Other:______________  99=No Response |  |
| 63. How often do you treat your drinking water?  **(Circle one)** | 0=Always  1=Sometimes  2=Never  99=Don’t Know/No Response |  |
| 64. Do you have an outside faucet? (**Circle one)** | 0=No  1=Yes  99=Don’t Know/no response |  |

| **Sanitation Survey Baseline**  **“*Now I would like to ask you a few questions about your sewer system.”*** | | |
| --- | --- | --- |
| 65. Where does the waste from your toilet go?  (**Read all the options and circle only one)** | 0=Septic tank/system  1=City/County Sewer System  2=Cesspool  3=To Ground, Extension pipe (no system)  4=Other, Explain:_______  99=Don’t Know/No Response | If 1,3,4,5, Skip to END  If 2, Skip to 72 |
| 66. Do you know what year your septic system was installed? | Year:______  99=Don’t Know/No Response |  |
| 67. Do you know if it was installed before or after you moved into this home?  (**Read all the options and circle only one)** | 0=Installed before I lived in this home  1=Installed when or after I moved to this home  99=Don’t Know/No Response |  |
| 68. When was the last time your septic system was serviced?  (**Read all the options and circle only one)** | 0=0-6 months ago  1=7-12 months ago  2=1-2 years ago  3=2-4 years ago  4=more than 4 years ago  99=Don’t Know/No Response |  |
| 69. Have any of the following been performed on your system since you lived here?  **(Read all the options mark 01, 00 or 99 for each choice).** | **0 =No, 01=Yes, 99=Don’t Know/No Response**  a. Tank Pumped  Year:________  b. Filter Cleaned  c. Sewer Pipes Replaced  d. Field lines replaced  e. System or Parts of System Replaced  Explain:_____________________ |  |
| 70. How many bedrooms do you have in your home? | 1. 3 or less  2. 4  3. 5 |  |
| 71. How many bathrooms do you have in your home? | 1. 1  2. 2.  3. 3  4. 4 or more |  |

**Is it ok for me to get a sample from one of your inside faucets?

**Is there an area I can use for about 5-10 minutes?

| “***Now I would like to take a look at your septic system to help me determine if it is working properly. Can you show me the septic tank or point me in the right direction?”*** | | |
| --- | --- | --- |
| 72. **(Observation only, do not ask, mark 01, 00 or 99 for each choice).**  Determine whether the septic system is working properly. Do you observe any of the following scenarios? | **00 =No, 01=Yes, 99=Don’t Know/No Response**  a. Surfacing sewage in the drain field area  b. Plumbing or septic tank backups  c. Slow draining fixtures  d. Gurgling sounds in the plumbing system  e. Sewage odors in the house or yard  f. Lush green grass over the drain-field  Comments: __________________________________ |  |
| 73. **(Observation only, Do not ask mark 01, 00) Take photograph of the location.**  Does the sewage pipe discharge to the ditch or the ground? | **00=No, 01=Yes,**   1. Wet area in the drain field 2. Straight pipe to ditch 3. Sewage odor 4. Un-mowed area |  |
| 74. **(Observation Only, Do Not Ask, mark 01, 00)**  Does the sewage pipe discharge to a water body, wetland or pond? | **00=No, 01=Yes,**   1. Straight pipe to water 2. Sewage odor in the yard |  |
