# Supplementary material for: Associations between Self-Reported Gastrointestinal Illness and Water System Characteristics in Community Water Supplies in Rural Alabama: A Cross-Sectional Study
Source: PLoS One. 2016 Jan 28;11(1):e0148102. doi: 10.1371/journal.pone.0148102 (PMC4731071; doi:10.1371/journal.pone.0148102)
Supplement: S1 Table — (DOCX) [file pone.0148102.s003.docx]

**Table: Descriptive List of Variables from Cross-Sectional Study on Water Supply and Health in Rural Alabama 2012**

| **Variable description** | **Format** |
| --- | --- |
| **Household level data** | **(as reported by the primary respondent)** |
| Rents home | Binary, Reference category was owning the home |
| College graduate in the home | Binary, reference category was no household member had a college degree |
| Connected to sewer | Binary, reference category was not being connected to sewer |
| Treating tap water | Binary, reference category was not treating tap water for drinking |
| Drinking bottled water | Binary, reference category was not reporting using bottled water for drinking regularly |
| Intermittent service | Binary, reference category was never reporting this condition |
| Low water pressure | Binary, reference category was never reporting this condition |
| Odd taste | Binary, reference category was never reporting this condition |
| Odd smell | Binary, reference category was never reporting this condition |
| Odd color | Binary, reference category was never reporting this condition |
| **Water quality** | **(as measured by interview staff at the household or the laboratory)** |
| Log_10_ Pressure | Continuous, log base 10 transformed PSI units where one unit increase represent a 10 fold increase in pressure |
| Turbidity | Binary, reference category is measured turbidity was <0.3 NTU |
| Free chlorine | Binary, reference category is free chlorine was <0.1mg/L |
| Total chlorine | Binary, reference category is total chlorine was <0.1mg/L |
| Outside tap – total coliforms | Binary, reference category is total coliforms < 1/100mL |
| Kitchen tap – total coliforms | Binary, reference category is total coliforms < 1/100mL |
| Outside tap – *E. coli* | Binary, reference category is *E. coli* < 1/100mL |
| Kitchen tap – *E. coli* | Binary, reference category is *E. coli* < 1/100mL |
| **Individual level data** | **(as reported by primary respondent)** |
| Age | Continuous, Age in years |
| Age (binary) | Binary, reference category is < 5 years of age |
| Sex | Binary, reference category is male |
| Race | Binary, reference category is white |
| Any symptoms | Binary, reference category is no reports of watery diarrhea, soft diarrhea, vomiting, nausea, or abdominal cramps. |
| Any vomiting | Binary, reference category is no report of vomiting |
| Any diarrhea | Binary, reference category is no report of water or soft diarrhea |
| AGI | Binary, reference category is no vomiting, no diarrhea (unless fewer than 3 stool in 24 hours)  Excludes individuals who have the following reported conditions: those with irritable bowel syndrome, Crohn’s disease, ulcerative colitis, celiac disease, and pregnancy |
